# Sizing biological cells using a microfluidic acoustic flow cytometer

**DOI:** 10.1038/s41598-019-40895-x

**Published:** 2019-03-18

**Authors:** Eric M. Strohm, Vaskar Gnyawali, Joseph A. Sebastian, Robert Ngunjiri, Michael J. Moore, Scott S. H. Tsai, Michael C. Kolios

**Affiliations:** 1 Department of Physics, Ryerson University, 350 Victoria St, Toronto, Canada; 2 Department of Mechanical and Industrial Engineering, Ryerson University, 350 Victoria St, Toronto, Canada; 3 Institute for Biomedical Engineering and Science Technology, a partnership between Ryerson University and St. Michael’s Hospital, M5B 1W8 Toronto, Canada; 4 Keenan Research Center for Biomedical Science, Li Ka Shing Knowledge Institute, St Michael’s Hospital, M5B 1W8 Toronto, Canada

## Abstract

We describe a new technique that combines ultrasound and microfluidics to rapidly size and count cells in a high-throughput and label-free fashion. Using 3D hydrodynamic flow focusing, cells are streamed single file through an ultrasound beam where ultrasound scattering events from each individual cell are acquired. The ultrasound operates at a center frequency of 375 MHz with a wavelength of 4 μm; when the ultrasound wavelength is similar to the size of a scatterer, the power spectra of the backscattered ultrasound waves have distinct features at specific frequencies that are directly related to the cell size. Our approach determines cell sizes through a comparison of these distinct spectral features with established theoretical models. We perform an analysis of two types of cells: acute myeloid leukemia cells, where 2,390 measurements resulted in a mean size of 10.0 ± 1.7 μm, and HT29 colorectal cancer cells, where 1,955 measurements resulted in a mean size of 15.0 ± 2.3 μm. These results and histogram distributions agree very well with those measured from a Coulter Counter Multisizer 4. Our technique is the first to combine ultrasound and microfluidics to determine the cell size with the potential for multi-parameter cellular characterization using fluorescence, light scattering and quantitative photoacoustic techniques.

## Introduction

Flow cytometry is a high throughput technique used to count, size, and/or sort cells. Common commercial systems can characterize thousands of cells per second using a variety of measurements, including electrical impedance, fluorescence, light scattering, optical imaging and/or cell mass^1–6^. Since the invention of flow cytometry in the 1960’s, high throughput cell characterization techniques have made a revolutionary impact in the fields of hematology, cancer and AIDS research, among others^7,8^.

Microfluidic technologies for flow cytometry of single cells are becoming increasingly popular due to their small device size, easy fabrication, and integration with a wide range of instrumentation and analytical tools^9–11^. Microfluidic-based cell counters and sorters use a variety of approaches to classify cells, including: optical imaging^12^, electrical impedance^13,14^, electrokinetics^15^, inertial forces^16^, surface acoustic waves^17–19^, acoustophoresis^20–22^, and magnetic agents^23^. Comprehensive review articles summarizing these technologies can be found in the literature^24–27^.

Many flow cytometry technologies can be used to count and sort cells, however only electrical impedance (e.g. the Coulter Counter) can determine the absolute size of cells with good accuracy. Flow cytometry that uses light scattering (e.g. FACS) can determine relative cell size populations, but the distributions are system dependent^28^; imaging flow cytometry (e.g. Imagestream) can have resolution limitations^29^. Systems that use dynamic light scattering, laser diffraction, or bulk acoustic scattering techniques (e.g. Malvern, Dispersion Technology) are based on bulk sample approximations and require prior knowledge of the optical and/or acoustic sample properties; they also cannot measure individual cells. Systems based on inertial, electrokinetic, acoustophoretics and surface acoustic waves are limited to sorting cells according to their size and/or density differences; they cannot determine the size of the cells on a cell-by-cell basis. Therefore, a method that can non-invasively count and size single cells on a cell by cell basis using a simple microfluidic system is highly desirable.

Ultrasound is non-invasive, non-destructive and label-free, and can be used to characterize biological tissues and materials. Recently, high frequency pulse echo ultrasound in the 20–60 MHz range has been used to quantify tissue properties based on underlying tissue structure and biomechanical properties to aid in the diagnosis of diseases, such as liver fibrosis and cancer^30–34^. While these ultrasound frequencies are appropriate for the assessment of bulk tissue properties, higher frequencies are required to probe individual cells. The theory which models the scattering of sound waves from spherical objects was first developed in the 1950’s^35^ and then refined over the next several decades; the scattering behavior is well established^36–39^. Using this scattering theory, we recently demonstrated that it is possible to determine the size of single cells using an acoustic microscope with ultrasound frequencies over 100 MHz^40^; however, this method was slow and laborious, requiring manual targeting of individual stationary cells, making it unsuitable for measuring large cell populations.

Conference papers published in 2014 described using custom designed microfluidic devices and quantitative pulse echo ultrasound techniques to determine the size of flowing 80 and 100 μm diameter microspheres using 30 MHz by Komatsu *et al*.^41^, and 6 and 10 μm diameter microspheres using 200 MHz by Strohm *et al*.^42^. These systems used a 3D flow focusing technique and compared the backscattered ultrasound power spectra from single microspheres to the Faran scattering model to determine the microsphere size. This demonstrated that for the first time, pulse echo ultrasound can be used to rapidly size flowing micro-sized particles; however, the frequencies were too low and thus lacked the spectral resolution to characterize cells.

Here, we describe the development of a high-throughput microfluidic-based acoustic flow cytometer that can be used to rapidly acquire ultrasound echoes from flowing single cells. We developed a novel 3D hydrodynamic flow focusing technique to stream cells within a 10 × 10 μm narrow path, integrated a high frequency ultrasound probe operating at 375 MHz, developed custom ultrasound hardware and software to rapidly insonify and acquire the ultrasound echoes from each passing cell, and applied a spectroscopic sizing algorithm to extract the size of each cell. At 375 MHz, the acoustic wavelength is 4 μm in water, which is slightly smaller than the size of a cell. In this scattering regime, ultrasound waves are preferentially scattered by the cell in different directions. The lack of specific frequencies in the backscattered ultrasound results in periodic minima throughout the power spectrum; these minima are directly related to the size of the cell. We demonstrate proof of concept using microspheres, and then analyze and two different cell lines with comparable size distributions predicted by the gold standard Coulter Counter. Our system is highly versatile and can be easily incorporated into a variety of microfluidic-based characterization systems to enable a multi-parameter analysis of each single cell using light scattering, fluorescence and photoacoustic techniques. Moreover, by inversion of the scattering problem, robust measurements of cell mechanical properties can be achieved.

## Results

### Microfluidic 3D flow focusing

**Figure 1:**
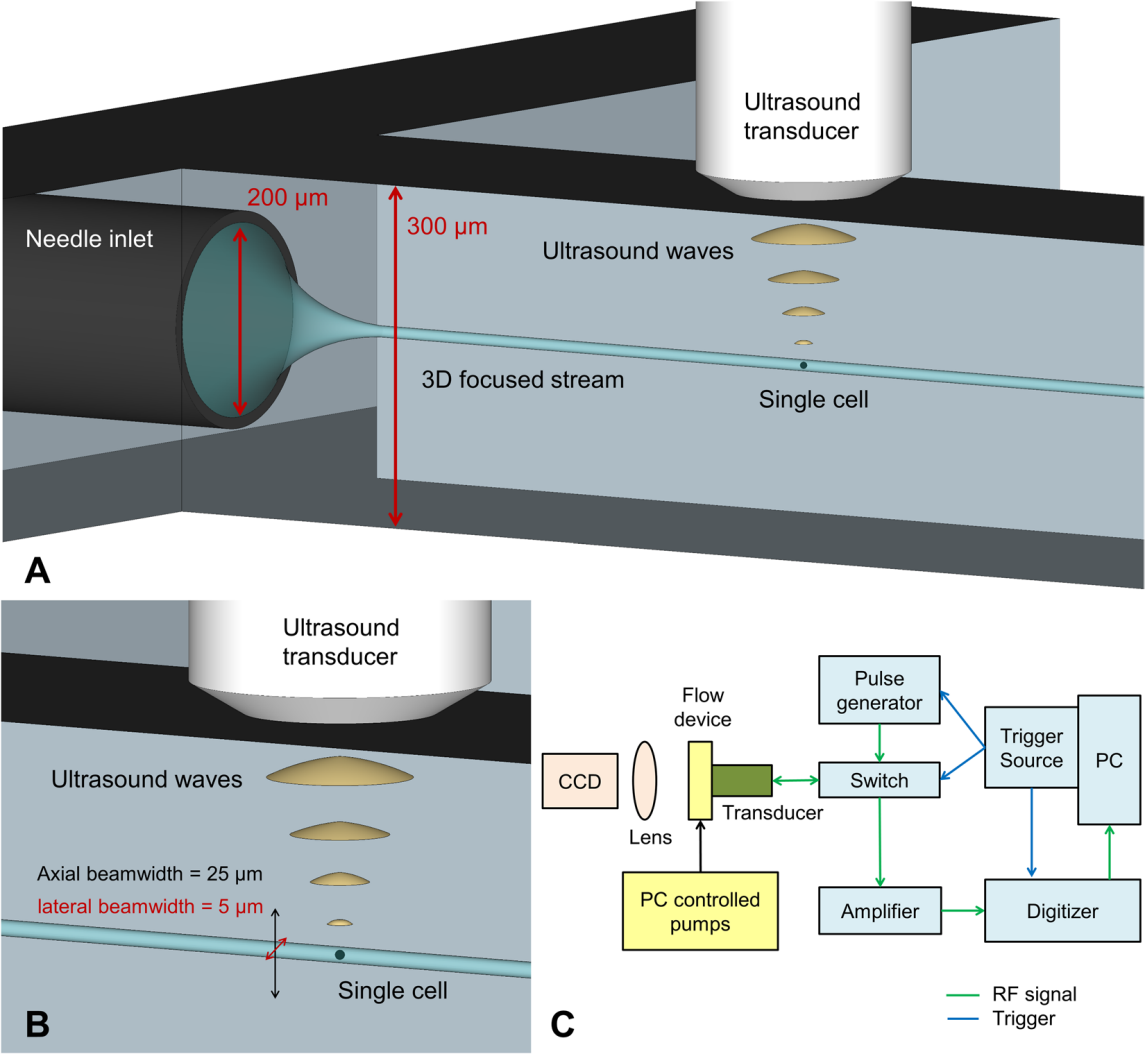
A schematic showing the acoustic flow cytometer concept. (**A**) Cells are hydrodynamically focused to a narrow stream in the lateral and axial directions. (**B**) The cells flow single file under the ultrasound transducer probe, where ultrasound scattered from each passing cell is recorded. The cells must pass through the narrow ultrasound beam, which has a beam width of approximately 5 × 25 μm (lateral & axial, respectively). (**C**) A schematic of the hardware developed to control the system and acquire the ultrasound signals.

**Figure 2:**
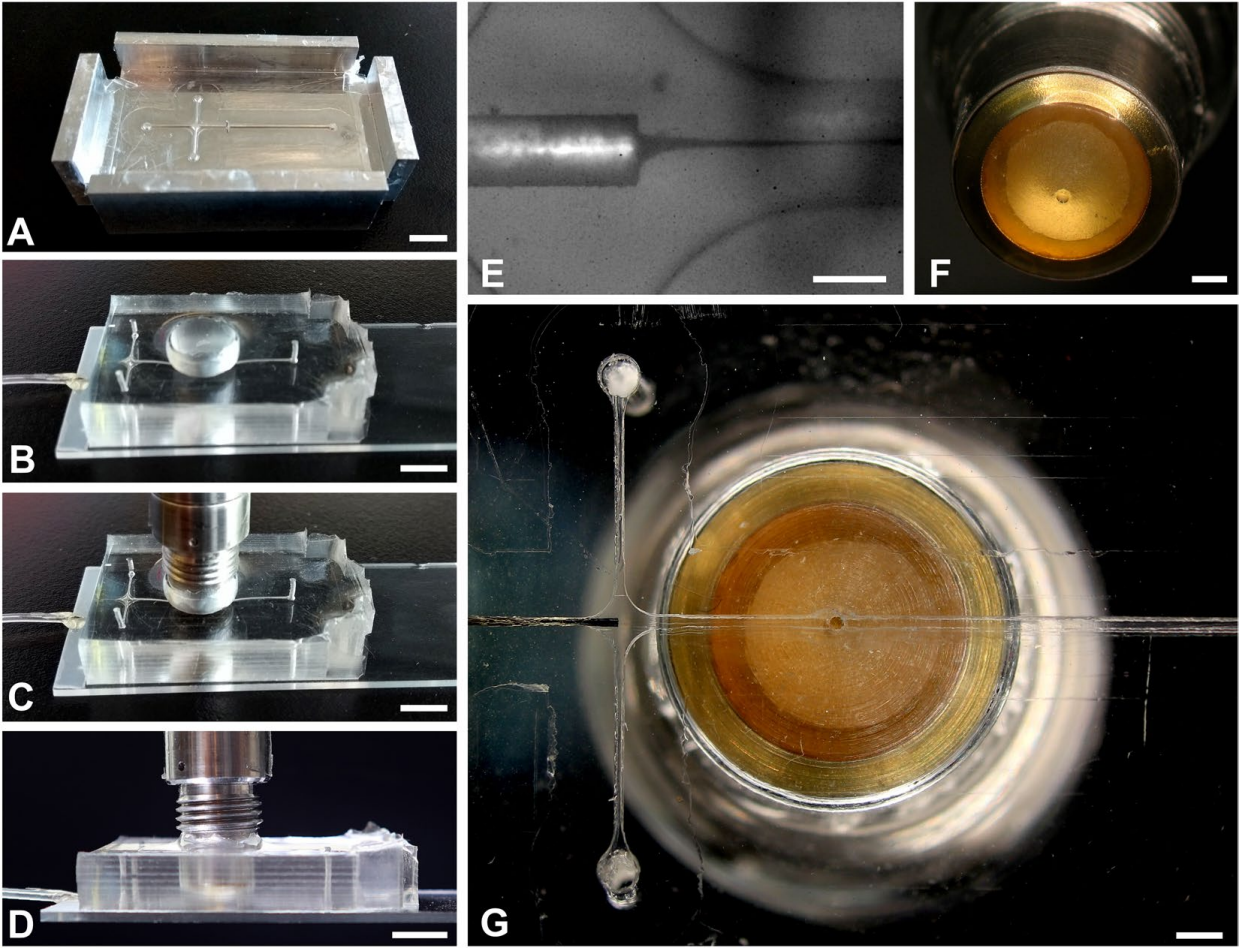
The PDMS flow focusing device. (**A**) The mold used to cast the PDMS. (**B–D**) The PDMS device bonded to the glass slide with the ultrasound transducer aligned to the focusing stream. (**E**) Optical image showing the flow focusing capability using black ink. (**F**) The ultrasound transducer, the aperture tip is 300 μm in diameter. (**G**) A bottom view of the PDMS device with the transducer aligned to the center of the flow channel. Scale bars: (**A–D**) 5 mm, (**E**) 200 μm, (**F,G**) 1 mm.

A high frequency ultrasound transducer was integrated into a polydimethylsiloxane (PDMS)-based microfluidic device to enable the rapid classification of cells using sound waves, as demonstrated by the schematic in Fig. 1A. The ultrasound beam has a lateral and axial field of view (FOV) of approximately 5 × 25 μm (Fig. 1B), and thus the flowing cells must be tightly focused in both the lateral and axial directions. To accomplish this, the PDMS microfluidic device was cast from a CNC-machined metal mold with a channel 300 μm in width and height. A second mold was used for a conical indentation for the ultrasound transducer tip (Fig. 2A–D). A 200 μm outer diameter needle was inserted at the inlet and positioned so that the sheath flow from the sides surrounded the needle. As a result, the stream from the needle inlet which carried the suspended cells was hydrodynamically focused to the center of the channel both laterally and axially (Fig. 2E). The center frequency of the transducer was 375 MHz with an aperture width and focal length of 300 μm (Fig. 2F), and was positioned axially so that the acoustic beam intersected the flowing cells (Fig. 2G). The lateral position of the cell stream was controlled by adjusting the relative flow rate of the two sheath flows. Custom designed hardware and software was used to insonify the stream of cells and digitize the back-scattered ultrasound signals (Fig. 1C).

### Validation using microspheres

**Figure 3:**
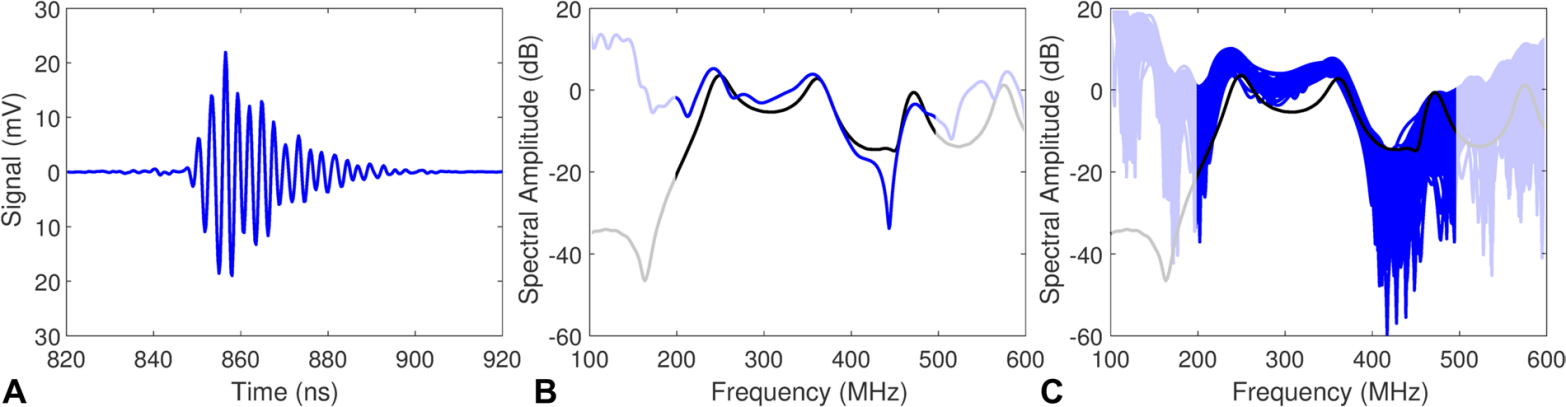
(**A**) The ultrasound signal measured from a single 3 μm polystyrene microsphere. (**B**) The measured ultrasound spectrum (blue) compared to the theoretical spectrum calculated using the Faran scattering model (black). (**C**) 150 consecutive measured spectra (blue) overlaid with the theoretical spectrum (black). Frequencies outside of the transducer bandwidth have been grayed.

Polystyrene microspheres were used to validate the system and analysis technique, as they are monodisperse and homogeneous, and the ultrasound spectra have well defined peaks at specific frequencies that are directly related to the size of the microsphere^43,44^. A solution of 3 μm white polystyrene microspheres in water was made at a concentration of 5–10 × 10^6^ microspheres/cm^3^ and flowed through the system. A representative measured time domain signal from a single 3 μm polystyrene microsphere is shown in Fig. 3A, along with the calculated power spectrum (Fig. 3B). The frequencies outside of the transducer bandwidth have been grayed to highlight higher signal to noise ratio (SNR) regions of the power spectrum. The theoretical ultrasound spectrum expected from the microsphere was calculated using the Faran scattering model^36,37^, where excellent agreement to the measured spectrum was observed. To illustrate the robustness of the measurement, the spectra from 150 consecutive microspheres were overlaid on a single figure (Fig. 3C). The 3 μm microsphere size was chosen as three separate spectral peaks occur over the transducer bandwidth that are easily discerned. As the microsphere size increases, the number of spectral peaks increases, and the peaks shift closer together. For microspheres larger than 6 μm, the spectral peaks merge and cannot be resolved at the 375 MHz frequencies used, and the model could not be used to identify the microspheres. While the composition and size of the microspheres differs from that of than cells, prior experience in using ultrasound scattering to characterize polystyrene microspheres has shown that the microspheres are suitable for validation of this high-throughput technique^43–47^.

### Cell measurements

**Figure 4:**
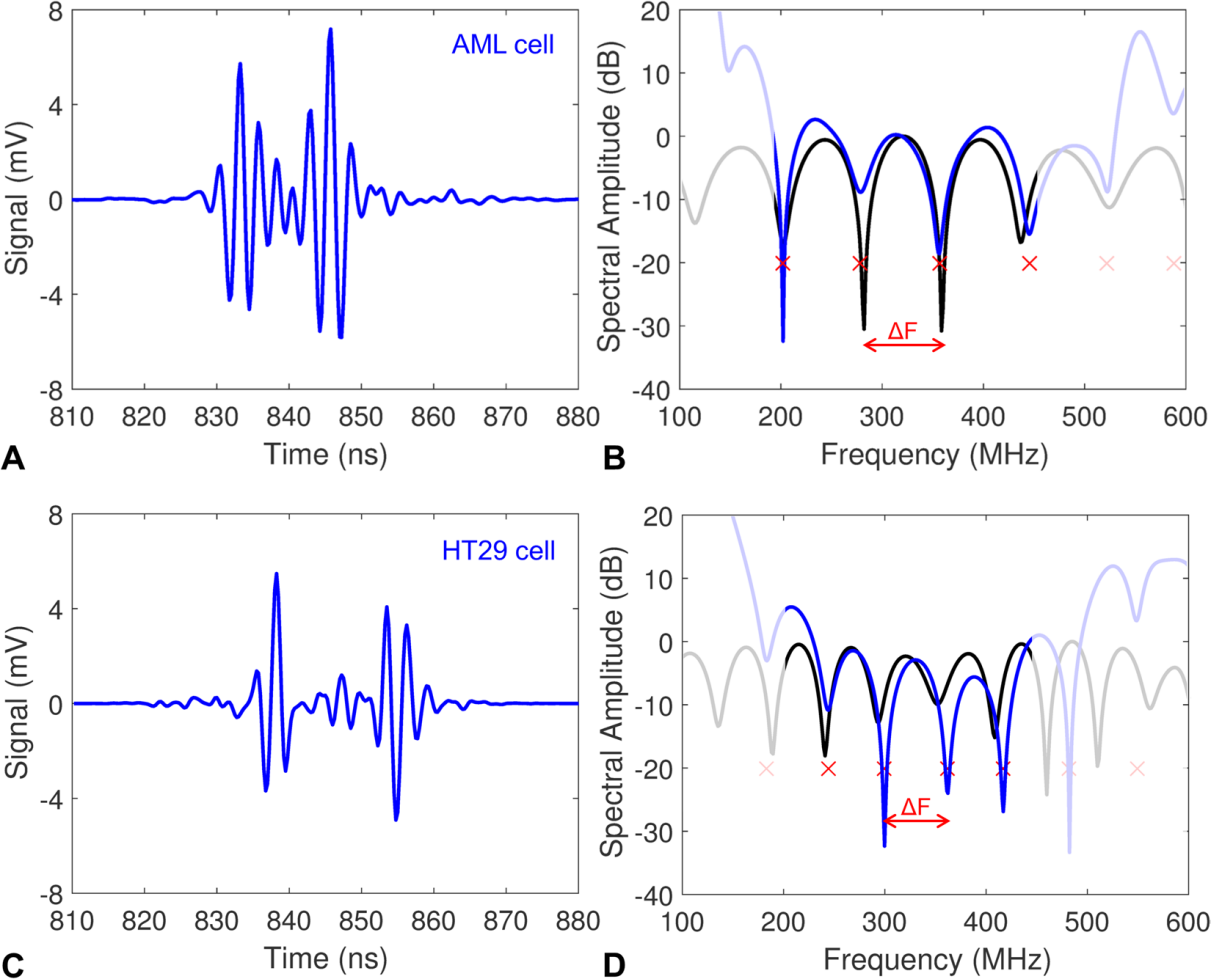
(**A**) A representative measured ultrasound signal from a single AML cell. (**B**) The measured ultrasound spectrum (blue) compared to the theoretical spectrum for a 10.2 μm diameter cell using the Faran scattering model (black). The red X’s indicate the spectral minima of the measured spectra using the peak detector algorithm, from which the spectral width ΔF was calculated. (**C**) A representative measured ultrasound signal from a single HT29 cell. (**D**) The measured ultrasound spectrum (blue) compared to the theoretical spectrum for a 15.2 μm diameter cell using the Faran scattering model (black). Frequencies outside of the transducer bandwidth have been grayed.

Two cell lines were used: (1) acute myeloid leukemia (AML) cells, and (2) HT29 colorectal cancer cells, which are larger than the AML cells. All cell suspensions were prepared at a concentration of 5 × 10^6^ cells/cm^3^. A representative time domain signal from AML and HT29 cells is shown in Fig. 4A,C, respectively. The measured signal from each cell has two peaks separated in time, which can be interpreted as the echo from the front and back of the cell. This occurs when the acoustic impedance between the cell and the surrounding fluid is similar^48^. An animation showing how the ultrasound signal amplitude and the calculated power spectrum changes as the cell passes through the ultrasound beam is shown in Movie S1. The calculated power spectrum for a representative AML and HT29 cells are shown in Fig. 4B,D, depicting distinct minima at specific frequencies that are related to the cell size. A peak detector algorithm was used to find the spectral minima (indicated by the red X’s in Fig. 4B,D) and the spectral spacing between minima ΔF.

**Figure 5:**
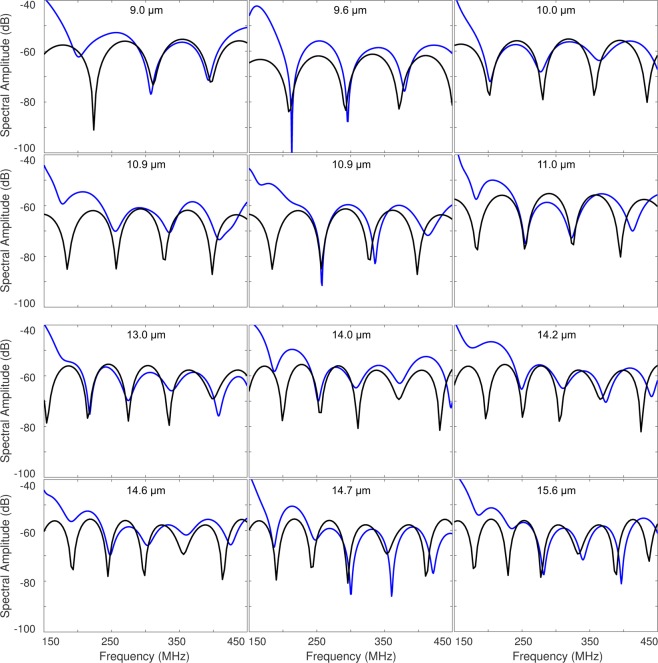
Typical measured signals from the AML and HT29 cells (blue) compared to the best fit model (black). The top two rows are AML cells, bottom two rows are HT29 cells.

**Figure 6:**
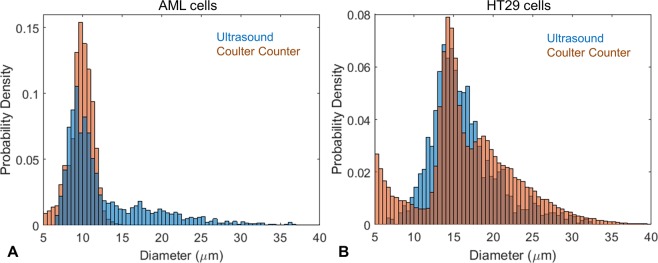
(**A**) Histogram showing the probability density function of the AML cell size measured using ultrasound (blue) and the Coulter Counter Multisizer 4 (orange). (**B**) Histogram showing the probability density function of the HT29 cell size measured using ultrasound (blue) and the Coulter Counter Multisizer 4 (orange).

A database of theoretical spectral data was calculated using the Faran scattering model for spheres with diameters ranging from 6 to 33 μm using a 0.1 μm step size, and the ΔF was calculated for each size. Values below 6 μm were not used as there was only one spectral minimum over the ultrasound bandwidth. The cell diameter was found by comparing the ΔF from the measured signal to the database values. The cell diameter associated with the ΔF that was closest to the measured value selected. The resulting best fit spectra for the representative AML and HT29 signals are shown in Fig. 4B,D, respectively. Typical spectra from the AML and HT29 cells are shown in Fig. 5. Spectra that had less than two spectral minima, or where the standard deviation in the ΔF calculation was greater than 25 MHz were discarded. A total of 6794 AML cells and 3420 HT29 cells were measured; after applying our selection criteria, 2390 measurements of AML cells were used to calculate a mean cell diameter of 10.0 ± 1.7 μm, while 1955 measurements of HT29 cells were used to calculate a mean cell diameter of 15.0 ± 2.3 μm. These results agreed with measurements using a Coulter Counter Multisizer 4, which measured a mean diameter of 9.8 ± 1.5 μm and 15.1 ± 2.2 μm for the AML and HT29 cells, respectively (Table 1). A histogram of the ultrasound and Multisizer 4 results in Fig. 6 shows the distribution of cell sizes, and further illustrates the good agreement between the ultrasound technique and the Multisizer, which is considered the gold standard for cell sizing. The mean diameter and standard deviation for both the Coulter Counter and Acoustic Flow Cytometry measurements were calculated over the range of 5–15 μm for AML cells, and 10–20 μm for HT29 cells. These ranges were chosen as they were centered around the peak diameter, and eliminated counts due to cellular debris (<6 μm) and clumping (>20 μm) that occurred for the HT29 cells.

**Table 1:** The size of the AML and HT29 cells measured using ultrasound and Coulter Counter.

Cell Type	Number of Measurements	Ultrasound Diameter (μm)	Coulter Counter Diameter (μm)
AML	2390	10.0 ± 1.7	9.8 ± 1.5
HT29	1955	15.0 ± 2.3	15.1 ± 2.2

## Discussion

The technique presented is the first to combine quantitative pulse-echo ultrasound and microfluidics to rapidly record ultrasound scattered from cells at high frequencies. At these frequencies, the wavelength of the ultrasound wave is on the order of the cell size, resulting in scattering patterns rich in features which are uniquely sensitive to the cell size. Biological cells are complex structures containing a nucleus, organelles and macromolecules, however they can be considered acoustically homogeneous at the ultrasound length scales used in this study. The measured ultrasound signals from the cells have two peaks separated in time (Fig. 4). When the acoustic impedance inside and outside the cell is similar, as it is in these studies, the two peaks that occur can be considered as the front and the back of the cell^48^. As the cell becomes larger, the time between the two peaks increases as it takes longer for the ultrasound to travel through the cell; this results in a decrease in the spectral width ΔF. This concept is illustrated in Fig. 4, where the time between peaks increased and the spectral width ΔF decreased between the AML cell (10.2 μm) and the HT29 cell (15.2 μm). Time domain methods can be used to calculate the cell size based on the time of flight of the signals^49^, however, this method is not as sensitive as the frequency domain analysis used here. The peak detector algorithm presented here was very simple yet effective; as larger volumes of data are generated, we will investigate deep learning and pattern matching algorithms to improve the classification technique as has been shown in optical scattering techniques.

The sizing algorithm relies on the comparison of measured spectra to theoretical predictions. Ultrasound scattering theory for spherical objects has been well described^35–39^. Additionally, our previous work demonstrates that the scattering models are robust in predicting the ultrasound signals and spectra of micrometer-sized polystyrene spheres, droplets and cells at ultra-high frequencies^40,43–46,49–51^. This is likely because cells in suspension tend to be spherical in shape, and when placed in a liquid solution, the interface with the greatest acoustic impendence mismatch at these frequencies is at the cell/solution interface. When the ultrasound wavelength is similar to the cell size, the density and sound speed of the scattering object dictate the spectral spacing, in addition to the size of the scatterer. The density and sound speed of the surrounding fluid (PBS or cell medium) are known (1000 kg/m^3^ and 1520 m/s, respectively), and the density and sound speed of the cells were estimated from previous studies measured using an acoustic microscope (1050 kg/m^3^ and 1560 m/s, respectively)^52,53^. With knowledge of these input parameters in the Faran model, the only unknown is the cell diameter. In our simulations, small changes in the cell density between the surrounding fluid and the cell do not affect the ultrasound spectral shape; the dominant factor that affects the frequencies of the spectral minima is the ratio of the cell sound speed to cell radius. We have previously measured the mechanical properties of single cells and found that the variation of the density and sound speed within a population of cells is only 1–2%, yet the cell size has a variation of over 50%^52,53^. Thus, while the sound speed can vary from cell to cell, it can be considered a known constant and the models are solved for the unknown cell size.

In an automated high throughput system, it is important to have consistent criteria for eliminating data for which single cell scattering does not apply. Cells clumping together tend to display more than two echoes in the time domain signal; cells flowing off-axis from the ultrasound beam tend to have one echo in the time domain signal, or the amplitude of the two echoes was significantly different. Signals that displayed these characteristics were eliminated from the data analysis. In our measurements of single stationary cells^40^, as the cell moved away from the center of the ultrasound beam, the SNR decreased and the spectral patterns were dominated by noise. This is noticeable in Movie S1, which shows the measured signal and power spectrum of a single cell as it passes through the ultrasound beam. The power spectral features are most prominent when the cell is in the middle of the beam, when the SNR is highest. We observed that more AML cell measurements were eliminated from the analysis compared to the HT29. This was likely due to the smaller AML size, as there is a greater probability that the larger HT29 cell would intersect the center of the ultrasound beam. To improve the SNR and reduce the chance of signals from smaller cells being eliminated from the analysis, we are investigating improved flow focusing methods, and better ways of reducing system noise, including increasing the signal averaging per cell and by increasing the ultrasound pulse repetition frequency (PRF) and using more sensitive transducers integrated directly into the PDMS device to enable larger amplitude pulse-echo signals.

Despite the numerous commercial and research-based microfluidic systems that have been developed that can differentiate, classify and sort cells, accurately determining the absolute size of individual cells remains a challenge. Aside from the Coulter Counter, all other flow cytometers cannot measure the absolute size of single cells accurately; some provide an estimate of the size distribution, while others can sort cells but not provide the cell size. This study demonstrates the proof of concept that ultra-high frequency ultrasound can be integrated into a microfluidic device, and that the scattered ultrasound can be used to determine the size distribution of micrometer-sized particles and cell populations on a cell by cell basis. The throughput can be improved through increasing the flow speed, ultrasound pulse repetition frequency and implementing parallel signal recording and analysis techniques for continuous operation. The microfluidic devices are easily fabricated, and the ultrasound hardware can be integrated into a wide variety of other microfluidic-based cell characterization systems, potentially enabling a multiparameter analysis of the cells to extract additional cellular details to further enhance cell classification. For example, ultrasound is sensitive to cell morphology^54,55^, a feature cannot be ascertained using traditional flow cytometry characterization systems. Adding a laser would enable simultaneous photoacoustic measurements. These simultaneous ultrasound and photoacoustic measurements would permit the label free calculation of the nucleus-to-cytoplasmic ratio of cells^51^, quantification of red blood cell morphology^56^, perform a blood count^57^, and could be used for other functional measurements of red blood cells^51,58^. Targeted nanoparticles^59^ or colorimetric dyes^60^ can be used to interrogate other aspects of cellular function. Fluorescent dyes could also be used in this set-up as most fluorescent dyes also generate strong photoacoustic signals. Finally, the cell mechanical properties can be measured with the appropriate solution to the inverse problem (such as the bulk modulus), allowing for another approach for measurements of the cell mechanotype for large cell populations.

## Conclusion

We have demonstrated that acoustic waves can be used to determine the size of cells in a high-throughput manner, with excellent agreement to the current standard, the Coulter Counter Multisizer. The custom designed PDMS device flow focused cells to a narrow stream using pressure driven pumps with excellent flow stability, while the integrated ultrasound transducer and hardware enabled rapid acquisition of high frequency ultrasound waves from each passing cell. Good flow stability with a narrow stream and fast ultrasound pulsing and acquisition with sub-nanosecond resolution were essential to measuring multiple signals per cell with good SNR. By comparing the measured ultrasound signals to known analytical solutions, we measured the cell size distribution of AML and HT29 cells, with a mean cell diameter of 10.0 ± 1.7 μm and 15.0 ± 2.3 μm, respectively. The mean size and standard deviation, as well as histogram shape were in good agreement with measurements from the Coulter Counter, validating the technique. Few methods exist that can determine the absolute size of cells on a cell by cell basis; in this study; we demonstrated that the high-throughput acoustic flow cytometer is highly sensitive to the cell size in the 6–30 μm range, and is ideally suited for measuring cell size distributions.

## Methods

### Microfluidic device

The microfluidic device was made of PDMS patterned using an aluminum mold. The microfluidic mold was made using a CNC-machined piece of aluminum with a raised ridge 300 μm in width and height for the flow channel (Fig. 2). The PDMS was poured onto the mold, and then another mold in the shape of the ultrasound transducer head was placed on top. The PDMS was left to cure for 3 hours at 70 °C. After curing, the PDMS chip was removed from the mold and then bonded onto a glass slide using oxygen plasma treatment. A 200 μm diameter needle was then inserted into one end of the device, and the ultrasound transducer inserted into the hole on the top (Fig. 2). Epoxy was used to seal the needle, and silicone was used to seal the transducer to the PDMS to prevent water leakage. The resulting device had three inlets, the center inlet containing the needle was for the sample flow, and the outer two inlets were for the sheath flow. A pressure pump (CorrSolutions, USA) was used to control the flow rates, where 1–5 μL/min was used for the cell flow, and 100–120 μL/min was used for the sheath flow. This resulted in the cells being hydrodynamically flow focused to the center of the channel laterally and axially with better stability than syringe pumps^61^. The optimal focusing position was found by starting with a small flow rate differential between the cell and sheath flow (e.g. 20 μL/min cell flow and 100 μL/min sheath flow), and shifting the flow laterally by gradually adjusting the relative flow rates of the two sheath flows. The axial position of the transducer was then adjusted to ensure the flow was in the center of the transducer axial FOV. Then, the cell flow rate was increased to create a narrow stream, and the stream was shifted laterally again by adjusting the relative sheath flow rates. This was repeated until a narrow stream was obtained. Once aligned, the system maintained good stability for over an hour.

### Ultrasound hardware and acquisition

We designed a custom ultrasound system to rapidly acquire the US signals from the cells flowing in the microfluidic device. An Intel i7 3770K-based computer with a trigger card (Spincore, USA) was used to control the hardware, and a Cobramax digitizer card (Gage Applied, USA) was used to acquire the signals using custom written software in Matlab (Mathworks, USA). A pulse generator (Geozondas, Lithuania) was used to generate monocycle pulses with a 0.5 ns pulse width and 15 Vpp amplitude to the US transducer. Ultrasound signals were sent through an RF-switch and a 100 MHz high pass filter (Mini-Circuits, USA), and then a 30 dB amplifier (Miteq, USA) before digitization (Fig. 1B). A train of 20 pulses at a PRF of 1 MHz was used to insonfiy the cells. The 20 pulses were averaged to increase SNR, as the cell would have negligible movement over the 20 μs duration of this pulse train. Each pulse train had a PRF of 2–5 kHz, which was dependent on the flow rate used, and chosen so that 10–15 signals per cell were acquired. The system acquired a batch 100 pulse trains at 4 GS/s, and then saved the signals only if a signal was greater than a 3 mV amplitude limit. This limit was imposed to achieve good SNR for the spectral analysis technique, as the noise floor of the system was around 1 mV. After each pulse train, there was a dead time of 2–3 ms while the system processed and saved the data. Future iterations will perform acquisition and analysis in parallel processes, significantly improving the throughput by increasing the number of cells that pass through the focal zone that are counted.

### Algorithms and analysis

All post processing was performed after data acquisition was complete. For each microsphere or cell signal, the pre-recorded background signal was subtracted, a Hamming window was applied and then the power spectrum was calculated using the Fast Fourier Transform. A reference spectrum was subtracted to remove the transducer response^43^. The microsphere spectra were compared directly to numerical simulations of ultrasound scattering from a 3 μm diameter polystyrene microsphere (2200 m/s sound speed, 1050 kg/m^3^ density and Poisson ratio of 0.35) using the Faran scattering model^36,37^. For cells, the diameter was unknown, and was found by comparing the measured spectra to simulations. This was done by finding the spectral minima using a peak detector algorithm (Matlab, USA) and then calculating the average spectral width ΔF. Results that had only one minimum, or where the standard deviation between minima was greater than 25 MHz were eliminated. The ΔF was compared to a database containing calculated theoretical ΔF values using the Faran scattering model where the cell diameter ranged from 6 to 33 μm (using a fixed 1560 m/s sound speed, 1050 kg/m^3^ density and a Poisson ratio of 0.499, with the assumption that the cells are incompressible). The cell diameter associated with that best fit model was then chosen as the cell diameter.

### Microsphere and cell suspensions

A microsphere suspension was made by diluting 3 μm microspheres (Polysciences, USA) into PBS to create a final concentration of 10 × 10^6^ cells/mL. AML and HT29 cells (ATCC, USA) were cultured in T75 culture flasks containing Dulbecco’s modified essential medium (DMEM) (for AML) or alpha-MEM (for HT29) with 10% FBS and 1% pen strep. Cells were passed every 2–3 days. Cells for the flow experiments were prepared by centrifuging the cells at 200 g for 10 minutes, and then diluted to a concentration of 5 × 10^6^ cells/mL using their respective cell culture media. Cell diameters were measured using a Coulter Counter Multisizer 4 (Beckham-Coulter, USA). HT29 cells were disassociated using trypsin. Alpha-MEM medium was added and the solution pipetted to reduce clumping. AML cells were diluted with DMEM medium prior to measurements. A 100 μm aperture tube was used.

## Supplementary information

Supplementary Information

Movie S1
