# Supplementary material for: Sizing biological cells using a microfluidic acoustic flow cytometer
Source: Sci Rep. 2019 Mar 18;9:4775. doi: 10.1038/s41598-019-40895-x (PMC6423196; doi:10.1038/s41598-019-40895-x)
Supplement: Supplementary file 1 — Supplementary Information [file 41598_2019_40895_MOESM1_ESM.doc]

# Sizing biological cells using a microfluidic acoustic flow cytometer

Eric M. Strohm^1,3,4^, Vaskar Gnyawali^2,3,4^, Joseph A. Sebastian^1,3,4^, Robert Ngunjiri^1,3,4^, Michael J. Moore^1,3,4^, Scott S. H. Tsai^2,3,4^, Michael C. Kolios*^1,3,4^

^1^ Department of Physics, Ryerson University, 350 Victoria St, Toronto, Canada,

^2^ Department of Mechanical and Industrial Engineering, Ryerson University, 350 Victoria St, Toronto, Canada

^3^ Institute for Biomedical Engineering and Science Technology, a partnership between Ryerson University and St. Michael’s Hospital, Toronto, Canada, M5B 1W8

^4^ Keenan Research Center for Biomedical Science, Li Ka Shing Knowledge Institute, St Michael’s Hospital, Toronto, Canada, M5B 1W8

*mkolios@ryerson.ca

# Supporting Information

**Movie S1**: Animation showing the measured time domain signal and power spectrum as a single cell passes through the ultrasound beam. The time between frames is 250 μs.
