# Supplementary figures and images for: Sizing biological cells using a microfluidic acoustic flow cytometer
Source: Sci Rep. 2019 Mar 18;9:4775. doi: 10.1038/s41598-019-40895-x (PMC6423196; doi:10.1038/s41598-019-40895-x)

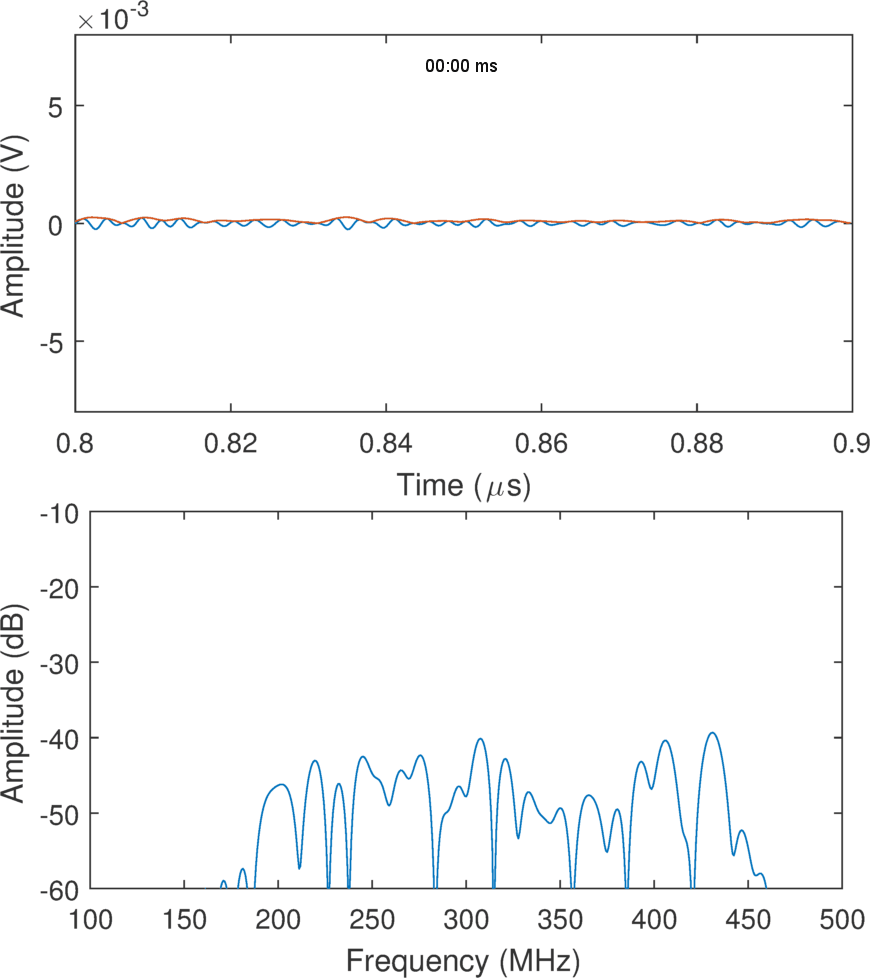

Supplement: Supplementary file 2 — Movie S1 [file 41598_2019_40895_MOESM2_ESM.gif]
